# Dexmedetomidine in The Treatment of Toxicologic Conditions in The Emergency Department: A Dual-Center Retrospective Observational Cohort Study

**DOI:** 10.1007/s13181-026-01145-5

**Published:** 2026-07-10

**Authors:** Kevin Baumgartner, Amar Chakraborty, David B. Liss, Sarah Berg, Miya Smith, Taylor Kaser, Rachel Ancona, Julianne C. Yeary, Samantha C. Lee, Jon B. Cole

**Affiliations:** 1 Division of Medical Toxicology, Department of Emergency Medicine, Washington University School of Medicine, St. Louis, MO USA; 2 Minnesota Regional Poison Control Center, Minneapolis, MN USA; 3 British Columbia Drug and Poison Information Centre, Vancouver, BC Canada; 4 Department of Emergency Medicine, Washington University School of Medicine, St. Louis, MO USA; 5 Barnes-Jewish Hospital, St. Louis, MO USA; 6 Division of Medical Toxicology, Department of Emergency Medicine, Hennepin Healthcare, Minneapolis, MN USA

**Keywords:** Dexmedetomidine, Emergency department, Intoxication, Poisoning

## Abstract

**Introduction:**

Dexmedetomidine has been used to treat toxicologic conditions, but high-quality data on its use for this indication in the emergency department setting are lacking. We sought to characterize the use of dexmedetomidine in this context.

**Methods:**

We conducted a dual-center retrospective cohort study of patients treated with dexmedetomidine in two academic emergency departments. We included patients who received dexmedetomidine in the emergency department and had a toxicologic condition, as adjudicated by a medical toxicologist on chart review. We abstracted data from the electronic medical record. We conducted multilevel logistic regression to explore factors associated with intubation after the initiation of dexmedetomidine.

**Results:**

We screened 1,081 patients and included 320. The most common toxicologic conditions were ethanol withdrawal (20%) and acute poisoning by sympathomimetics (19.7%). Culprit xenobiotics were primarily ethanol and substances of misuse. Median nadir heart rate and mean arterial pressure after dexmedetomidine initiation were normal, and there was no substantial change in nadir hemodynamic parameters after dexmedetomidine administration, although there was an increase in vasopressor administration (6.6% to 11.9%). Among patients who were not intubated when dexmedetomidine was initiated (*n* = 200), the frequency of intubation was 21.5% (95% CI 16–28). On multilevel logistic regression, antipsychotic administration was associated with increased odds of intubation (adjusted odds ratio 2.52, 95% CI 1.15–5.51).

**Conclusion:**

Dexmedetomidine was primarily used to treat emergency department patients with intoxication or withdrawal from ethanol and substances of misuse. The frequency of intubation in spontaneously breathing patients after the initiation of dexmedetomidine was 21.5%. Further prospective research is needed to evaluate dexmedetomidine in the treatment of toxicologic conditions.

**Supplementary Information:**

The online version contains supplementary material available at 10.1007/s13181-026-01145-5.

## Introduction

Dexmedetomidine (DEX) is a centrally acting sympatholytic [1] which is used as a sedative in many clinical contexts, including mechanical ventilation [2], non-invasive positive pressure ventilation [3], and procedural sedation [4]. DEX produces a unique state of “cooperative sedation" [5] which allows sedated patients to cooperate in their care and communicate with medical staff [6], and does not appear to depress the respiratory drive at usual doses [1]. The mechanism of action of DEX, which agonizes presynaptic alpha-2 adrenoceptors and imidazoline receptors [1, 7], is distinct from the mechanism of other common sedating medications, and may be useful in directly countering excessive sympathetic tone. These properties make DEX an attractive option for the sedation of patients with toxicologic conditions.

The use of DEX in toxicologic conditions has been reported, but the published evidence is heterogeneous and of generally poor quality, consisting almost entirely of case reports and case series [8]. Previous observational cohort studies have been limited by small sample sizes, single-center designs, and missing or non-granular data on coadministration of other sedatives [9–11]. Additionally, the use of DEX in toxicologic conditions specifically in the emergency department (ED) has not been described in detail outside of case reports, as many previous studies have focused on intensive care unit (ICU) patients or not distinguished among treatment settings. This is consistent with the paucity of high-quality evidence on the use of DEX for *any* purpose in the ED [12]. Given the differences in patient population, stage of resuscitation and diagnostic workup, staffing, and cultural standards between the ED and ICU, ED-specific evaluation of critical care interventions and practices is an important component of translation research.

In order to address this knowledge gap, we conducted a dual-center retrospective observational cohort study of the use of DEX in the treatment of toxicologic conditions in the ED.

## Methods

### Study Design and Reporting

We conducted a dual-center retrospective observational cohort study of patients treated for toxicologic conditions with DEX in the ED. This study was approved by the Washington University School of Medicine & Hennepin Healthcare Institutional Review Boards (IRB # 202,407,193 and #FY2024-090, respectively). We conducted and reported the study in adherence to the Strengthening Reporting for Observational Studies in Epidemiology (STROBE) guidelines [13]. A completed STROBE checklist is presented in Appendix 1.

### Population and Setting

We identified patients for inclusion from two hospitals: Barnes-Jewish Hospital (BJH), a quaternary academic hospital with an annual ED census of approximately 80,000 in St. Louis, Missouri, and Hennepin County Medical Center (HCMC), a safety-net, county hospital with an annual ED census of approximately 95,0000 in Minneapolis, Minnesota. The study period was from the inception of the current electronic medical record (EMR; January 2007 for HCMC and June 2018 for BJH) through November 2024. All patients who received intravenous DEX in the ED of BJH or HCMC during the study period were identified via EMR query and screened for inclusion.

### Inclusion and Exclusion Criteria

We included patients who were treated with DEX by intravenous infusion in the ED at BJH or HCMC during the study period and who had a toxicologic condition, in the judgment of an attending medical toxicologist (see below). We excluded patients with missing data on sedative administration in the ED.

### Research Objectives and Outcomes

Our primary objective was to characterize the use of DEX for toxicologic conditions in the ED. We aimed to describe indications for DEX, clinical characteristics of patients receiving DEX, dosing of DEX and co-administration of other sedatives, and hemodynamic effects of DEX. Our secondary objective was to calculate the frequency of intubation following the administration of DEX for toxicologic conditions in the ED and to evaluate factors associated with this outcome.

### Study Protocol

We generated lists of patients who received intravenous DEX in the ED during the study period via EMR query. Identifying information for these encounters (encounter number, medical record number, date of service, and hospital) were imported into a pre-built, secure, standardized electronic database (Research Electronic Data Capture, REDCap [14, 15]). Encounters were then randomly assigned to a single attending medical toxicologist (DBL, SB, or MS for BJH; JBC for HCMC) for review. Reviewing medical toxicologists underwent standardized training on study procedures, including the use of the REDCap instrument. They reviewed the totality of data for each ED encounter (clinician notes, medication administration, and diagnostics results) in the local EMR and determined the presence or absence of a toxicologic condition, defined broadly to include any acute or chronic poisoning, adverse drug effect, withdrawal syndrome, intoxication, or envenomation. If they judged that a toxicologic condition was present, they entered the pre-defined type of condition (acute poisoning/intoxication, chronic poisoning/intoxication, withdrawal syndrome, adverse drug event, envenomation, mixed presentation [e.g. both intoxication and withdrawal were present in the same encounter], or other) and categorized the toxidrome(s) and culprit agent(s) according to the definitions used by the Toxicology Investigators Consortium (ToxIC) registry [16]. Reviewing toxicologists could indicate that a single culprit agent was primarily responsible for the patient’s presentation or that multiple agents contributed. We did not segregate patients by intentionality of the exposure.

All patients adjudicated to have a toxicologic condition were included in the study. Data abstractors (KB, an attending medical toxicologist, for BJH; AC, a medical toxicology fellow, or SCL, a pharmacist and clinical toxicologist, for HCMC) then used a separate REDCap data collection instrument to abstract information of interest from the local EMR. Abstractors underwent standardized training on study procedures and definitions, including the use of the REDCap instrument. Data were abstracted on demographics, vital signs, dosing and administration of DEX and other sedatives, dosing and administration of vasopressors/inotropes, toxicologic interventions (as defined and categorized by the ToxIC registry [16]), mechanical ventilation and non-invasive positive-pressure ventilation, restraint use, ED sedation depth (Richmond Agitation-Sedation Scale [17] [RASS] and/or Glasgow Coma Score [18] [GCS]), diagnostic data (electrocardiogram [EKG] and select laboratory data), length of stay (ED, intensive care unit [ICU], hospital), hospital mortality, and continuation of DEX use after ED departure. All variables abstracted were objectively present in the EMR as discrete data. Vital signs, medications, assessments, and interventions were categorized as pre-DEX (any time prior to the initiation of DEX) and post-DEX (any time between the initiation of DEX and departure from the ED). Benzodiazepine doses were converted to milligram lorazepam equivalents [19], and vasopressor and inotrope infusion rates were converted to norepinephrine equivalents [20]. Drug testing panels, practices, and techniques varied throughout the study period at both sites. Urine drug testing was reported as positive if the substance of interest was detected on either immunoassay or chromatography-mass spectrometry. Corrected QT intervals were reported as per the computerized calculation on the EKG printout, and dysrhythmia risk was determined by evaluating paired heart rates and raw QT intervals using a linear approximation [21] of the QT nomogram [22]. Additional information on selected variable definitions is presented in Appendix 2.

### Statistical Analysis

We reported categorical variables as counts and frequencies and continuous variables as medians with interquartile ranges, as all distributions were non-normal. For our secondary objective, we characterized patients as intubated *prior to* DEX administration (not at risk of the outcome), intubated in the ED *after* DEX administration (experienced the outcome), and not intubated in the ED *after* DEX administration (did not experience the outcome). We assessed variables and outcomes for the full cohort and stratified by study site (BJH or HCMC), pre- or post-DEX timing, and/or intubation status (intubated prior to DEX, intubated after DEX, not intubated after DEX). Missing data were excluded from each respective variable for the descriptive analysis; no imputation was performed. We compared medians using Mann–Whitney U tests and counts and frequencies using Pearson's chi-square test or Fisher's exact test, as appropriate.

We conducted multilevel logistic regression to evaluate the association between clinical factors and intubation after DEX. Only patients at risk for the outcome (that is, not intubated prior to DEX administration) were included in this analysis. Factors evaluated were selected a priori, based on pharmacologic principles and clinical gestalt, and included blood ethanol concentration (detected, not detected, not tested), ED administration of any benzodiazepine while at risk for intubation, ED administration of any antipsychotic while at risk for intubation, and ED administration of ketamine (other than for rapid sequence intubation) while at risk for intubation. The primary model included fixed effects for the candidate factors. We reported odds ratios (OR) with 95% confidence intervals (95% CI) for the fixed effects. We assessed model fit using the marginal and conditional R^2^ and evaluated the intraclass correlation coefficient (ICC) to determine what proportion of total variance was explained by differences between study sites compared to differences in individuals within the sites. We conducted three sensitivity analyses: 1) adding a random effect for study site, 2) dropping patients not tested for ethanol and treating ethanol concentration as a continuous variable, and 3) extending the definition of "intubation" to include not just intubation in the ED, but *any* intubation in the 24 h following DEX initiation (e.g. later intubation in the ICU).

The sample size of this retrospective study was fixed by the number of patients meeting inclusion criteria during the study period. Based on prior work on DEX use in the ED [12, 23, 24] and investigator judgment, we expected to identify approximately 80 patients meeting inclusion criteria. Given the paucity of data on DEX in the ED for toxicologic conditions, we felt that this expected sample size was adequate to provide valuable descriptive insights and hypothesis-generating results to inform future research.

## Results

**Fig. 1:**
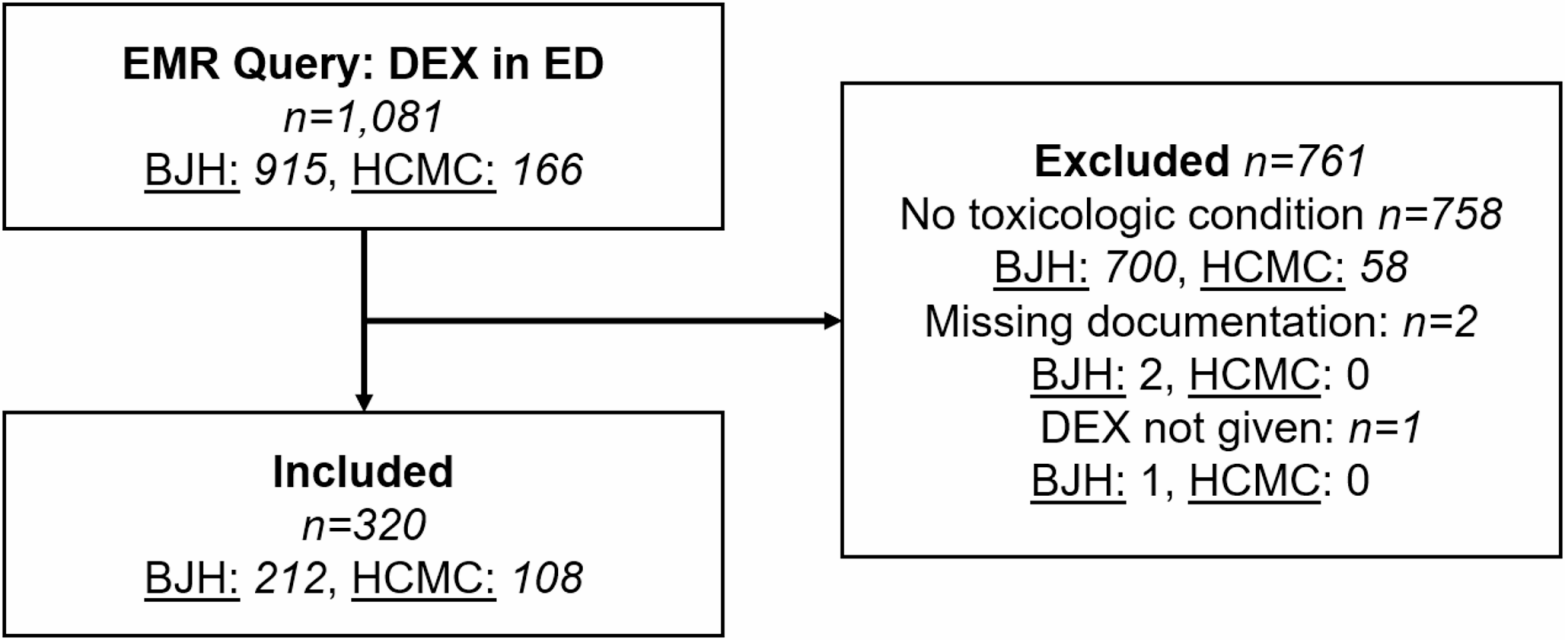
Patient flow diagram

We screened 1,081 patients and included 320, with 212 patients from BJH and 108 patients from HCMC. A patient flow diagram is presented in Fig. 1. Clinical characteristics, including demographics, study site, and toxicologic conditions, are presented in Table 1. Included patients were predominantly male (*n* = 245, 76.6%) and middle-aged (median age 45 year [IQR: 33—56]). The most common toxicologic conditions were ethanol withdrawal, acute poisoning by sympathomimetics, acute poisoning by multiple xenobiotic classes, and acute poisoning by ethanol. Conditions segregated by study site are presented in Appendix 3, and a comprehensive list of toxicologic conditions and xenobiotic classes is presented in Appendix 4.

**Table 1:** Patient characteristisc

	Full Cohort (*n* = 320)
Study site, *n* (%)	
BJH	212 (66.3%)
HCMC	108 (33.8%)
Age, years, median (IQR)*	45 (33—56)
Sex, n (%)	
Male	245 (76.6%)
Female	74 (23.1%)
Other	1 (0.3%)
Race, *n* (%)	
White	142 (44.4%)
Black	141 (44.1%)
American Indian or Native Alaskan	15 (4.7%)
Multiracial	4 (1.3%)
Unknown	18 (5.6%)
Ethnicity, *n* (%)	
Non-Hispanic	265 (82.8%)
Hispanic	9 (2.8%)
Unknown	46 (14.4%)
Body mass index, kg/m^2^, median (IQR)**	24.9 (22.4—29.3)
ED length of stay, h, median (IQR)	7.20 (4.90—11.4)
Toxicologic condition, *n* (%)	
Withdrawal, ethanol	64 (20%)
Acute poisoning, sympathomimetics	63 (19.7%)
Acute poisoning, multiple classes	55 (17.2%)
Acute poisoning, ethanol	52 (16.3%)
Other conditions	86 (26.9%)
Acute poisoning, other single class	53 (16.6%)
Withdrawal, other	14 (4.4%)
Adverse drug event	11 (3.4%)
Mixed presentation	8 (2.5%)
ED disposition, *n* (%)	
Intensive care unit	275 (85.9%)
Discharged	27 (8.4%)
Floor	14 (4.4%)
Stepdown unit	3 (0.9%)
Expired	1 (0.3%)
ED or hospital mortality, *n* (%)	8 (2.5%)

BJH = Barnes-Jewish Hospital, HCMC = Hennepin County Medical Center, *IQR* Interquartile range, *ED* Emergency department

^*^ Missing data for one patient whose identity was never determined

^**^ Missing data for 7 patients due to missing weight or height in the ED record

Treatments and diagnostics are presented in Table 2, segregated by toxicologic condition. Critical care interventions were common; 163 patients (50.9%) were intubated in the ED, and 55 (17.2%) received vasopressors. DEX was infused at low rates (median time-weighted average rate 0.34 mcg/kg/h, IQR 0.22–0.56) for brief periods (median DEX duration 2.45 h, IQR 1.4–4.2) and discontinued in the ED in about a third of cases. Co-administration of other sedatives was universal. Benzodiazepines (*n* = 223, 69.7%) and antipsychotics (*n* = 144, 45%) were the most common. There was not a consistent trend towards reductions in administration or dosing of other sedatives pre- and post-DEX. Restraint use was common (63%) and did not appear to differ before and after initiation of DEX. RASS and GCS data were frequently missing, particularly pre-DEX RASS and post-DEX GCS (see Table 2 caption). Additional data on sedative doses are presented in Appendix 5.

**Table 2:** Emergency department treatments and diagnostics

	EWD (*n* = 64)	AP-S (*n* = 63)	AP-M (*n* = 55)	AP-E (*n* = 52)	Others (*n* = 86)	Full Cohort (*n* = 320)
Initial DEX infusion rate, mcg/kg/h, median (IQR) *	0.3 (0.3, 0.3)	0.2 (0.2, 0.25)	0.2 (0.2, 0.3)	0.2 (0.2, 0.3)	0.2 (0.2, 0.3)	0.2 (0.2, 0.3)
Maximum DEX infusion rate, mcg/kg/h, median (IQR) *	0.6 (0.3, 1)	0.4 (0.25, 0.6)	0.5 (0.3, 1)	0.3 (0.2, 0.65)	0.5 (0.3, 0.7)	0.4 (0.3, 0.775)
Time-weighted average DEX infusion rate, mcg/kg/h, median (IQR) *	0.44 (0.3, 0.79)	0.29 (0.21, 0.435)	0.39 (0.265, 0.595)	0.285 (0.2, 0.543)	0.355 (0.203, 0.58)	0.34 (0.22, 0.555)
Time from ED presentation to DEX initiation, h, median (IQR)	5.6 (3.63, 8.3)	3.6 (1.45, 6.35)	2.5 (1.4, 6.15)	2.4 (1.5, 3.7)	2.6 (1.3, 4.85)	3.3 (1.5, 6.3)
DEX infusion duration, h, median (IQR)*	2 (1.1, 3.4)	2.4 (1.5, 4.3)	3.4 (1.7, 5.15)	2.35 (1, 4)	2.6 (1.5, 4.28)	2.45 (1.4, 4.2)
DEX discontinued in ED, *n* (%)*	13 (20.3%)	20 (31.7%)	18 (32.7%)	22 (42.3%)	25 (29.1%)	98 (30.6%)
Intubation status, *n* (%)						
Not intubated in ED	41 (64.1%)	34 (54.0%)	26 (47.3%)	20 (38.5%)	36 (41.9%)	157 (49.1%)
Intubated in ED prior to DEX administration	15 (23.4%)	18 (28.6%)	26 (47.3%)	28 (53.8%)	33 (38.4%)	120 (37.5%)
Intubated in ED after DEX administration	8 (12.5%)	11 (17.5%)	3 (5.5%)	4 (7.7%)	17 (19.8%)	43 (13.4%)
Sedative receipt, *n* (%)						
Benzodiazepines	62 (96.9%)	40 (63.5%)	42 (76.4%)	28 (53.8%)	51 (59.3%)	223 (69.7%)
*Pre-DEX*	60 (93.8%)	36 (57.1%)	37 (67.3%)	23 (44.2%)	45 (52.3%)	201 (62.8%)
*Post-DEX*	19 (29.7%)	17 (27%)	21 (38.2%)	16 (30.8%)	27 (31.4%)	100 (31.3%)
Antipsychotics	22 (34.4%)	33 (52.4%)	32 (58.2%)	23 (44.2%)	34 (39.5%)	144 (45%)
*Pre-DEX*	18 (28.1%)	29 (46%)	28 (50.9%)	20 (38.5%)	29 (33.7%)	124 (38.8%)
*Post-DEX*	5 (7.8%)	7 (11.1%)	8 (14.5%)	6 (11.5%)	9 (10.5%)	35 (10.9%)
Propofol	23 (35.9%)	24 (38.1%)	26 (47.3%)	24 (46.2%)	43 (50%)	140 (43.8%)
*Pre-DEX*	15 (23.4%)	16 (25.4%)	22 (40%)	17 (32.7%)	26 (30.2%)	96 (30%)
*Post-DEX*	23 (35.9%)	23 (36.5%)	25 (45.5%)	22 (42.3%)	37 (43%)	130 (40.6%)
Ketamine	1 (1.6%)	21 (33.3%)	19 (34.5%)	15 (28.8%)	28 (32.6%)	84 (26.3%)
*Pre-DEX*	1 (1.6%)	18 (28.6%)	15 (27.3%)	10 (19.2%)	21 (24.4%)	65 (20.3%)
*Post-DEX*	0 (0%)	6 (9.5%)	8 (14.5%)	7 (13.5%)	11 (12.8%)	32 (10%)
Fentanyl	6 (9.4%)	20 (31.7%)	12 (21.8%)	28 (53.8%)	34 (39.5%)	100 (31.3%)
*Pre-DEX*	4 (6.3%)	17 (27%)	11 (20%)	24 (46.2%)	22 (25.6%)	78 (24.4%)
*Post-DEX*	3 (4.7%)	14 (22.2%)	10 (18.2%)	23 (44.2%)	31 (36%)	81 (25.3%)
Phenobarbital	25 (39.1%)	2 (3.2%)	0 (0%)	1 (1.9%)	3 (3.5%)	31 (9.7%)
*Pre-DEX*	22 (34.4%)	2 (3.2%)	0 (0%)	1 (1.9%)	2 (2.3%)	27 (8.4%)
*Post-DEX*	6 (9.4%)	0 (0%)	0 (0%)	0 (0%)	1 (1.2%)	7 (2.2%)
ED RASS, median (IQR)***	1 (−1, 1)	1 (−1, 2)	1 (−2, 2)	1 (−2, 1)	1 (−1, 2)	1 (−1, 2)
Pre-DEX	1 (0, 1)	1 (−2, 1)	−2 (−3, 1)	−2 (−2, −1)	1 (−2, 1)	0 (−2, 1)
Post-DEX	0 (−2, 1)	0 (−1, 1)	−1 (−2, 1)	0 (−2, 1)	1 (−2, 2)	0 (−2, 2)
ED GCS, median (IQR)****	14 (13, 15)	14 (10, 15)	11 (7, 14)	10 (4, 14)	13 (9, 15)	13 (9, 14)
Pre-DEX	14 (13, 15)	13 (9, 15)	12 (7, 14)	10 (3, 14)	14 (10, 15)	13 (9, 14)
Post-DEX	14 (11, 15)	14 (10, 15)	10 (7, 14)	12 (11, 14)	12 (8, 15)	12 (9, 15)
Any restraint use, *n* (%)	33 (51.6%)	40 (63.5%)	44 (80%)	34 (65.4%)	51 (59.3%)	202 (63.1%)
Pre-DEX	30 (46.9%)	35 (55.6%)	35 (63.6%)	28 (53.8%)	37 (43%)	165 (51.6%)
Post-DEX	30 (46.9%)	38 (60.3%)	42 (76.4%)	33 (63.5%)	49 (57%)	192 (60%)
Restraint duration, h, median (IQR)**	6.2 (3.5, 7.4)	5.5 (3.83, 8.05)	5.75 (3, 8.4)	4.55 (2.85, 5.98)	4.25 (1.9, 6)	5.3 (3, 7.4)
Pre-DEX	3.2 (0.8, 5.7)	1.7 (0.475, 4.8)	1.45 (0.475, 4.58)	1.2 (0.3, 2.53)	0.45 (0, 2.55)	1.3 (0.3, 3.8)
Post-DEX	1.8 (1.1, 3.4)	3.05 (1.55, 5.13)	3.9 (2.23, 5.3)	2.6 (1.33, 5.15)	2.8 (1.63, 4.28)	2.8 (1.4, 4.8)
Any ED NPPV, *n* (%)	19 (29.7%)	16 (25.4%)	9 (16.4%)	4 (7.7%)	20 (23.3%)	68 (21.3%)
Pre-DEX	17 (26.6%)	15 (23.8%)	8 (14.5%)	4 (7.7%)	14 (16.3%)	58 (18.1%)
Post-DEX	9 (14.1%)	12 (19.0%)	6 (10.9%)	2 (3.8%)	18 (20.9%)	47 (14.7%)
Toxicologic intervention, *n* (%)						
Thiamine	47 (73.4%)	1 (1.6%)	3 (5.5%)	6 (11.5%)	2 (2.3%)	59 (18.4%)
Folate	40 (62.5%)	1 (1.6%)	2 (3.6%)	2 (3.8%)	0 (0%)	45 (14.1%)
Naloxone	0 (0%)	2 (3.2%)	9 (16.4%)	6 (11.5%)	14 (16.3%)	31 (9.7%)
Tested positive for ethanol, *n* (%)***	16 (25.0%)	5 (7.9%)	17 (30.9%)	50 (96.2%)	9 (10.5%)	97 (30.3%)
Ethanol, mg/dL, median (IQR)****	101 (31.3, 177)	64 (50, 67)	160 (134, 246)	259 (185, 309)	98 (27, 295)	196 (107, 295)
Urine drug screen positivity, *n* (%)	14 (21.9%)	51 (81%)	43 (78.2%)	29 (55.8%)	57 (66.3%)	194 (60.6%)
Fentanyl	1 (1.6%)	27 (42.9%)	28 (50.9%)	14 (26.9%)	41 (47.7%)	111 (34.7%)
Benzodiazepines	7 (10.9%)	21 (33.3%)	22 (40%)	17 (32.7%)	20 (23.3%)	87 (27.2%)
Cocaine	1 (1.6%)	31 (49.2%)	21 (38.2%)	2 (3.8%)	17 (19.8%)	72 (22.5%)
Cannabinoids	3 (4.7%)	16 (25.4%)	9 (16.4%)	19 (36.5%)	20 (23.3%)	67 (20.9%)
Amphetamine	0 (0%)	20 (31.7%)	20 (36.4%)	2 (3.8%)	14 (16.3%)	56 (17.5%)

Only the top three toxicologic interventions by frequency are shown. Urine drug screen positivity indicates that the substance was detected *either* by immunoassay *or* by chromatography/mass spectrometry. Ethanol positivity results exclude *n* = 102 patients not tested for ethanol, and ethanol concentrations include only patients with detectable ethanol

*DEX* Dexmedetomidine, *EWD* Ethanol withdrawal, *AP-S* Acute poisoning by sympathomimetics, *AP-M* Acute poisoning by multiple substance classes, *AP-E* Acute poisoning by ethanol, *IQR* interquartile range, *ED* Emergency department

^*^ Excludes one EWD patient from HCMC who only received DEX via bolus

^**^ Excludes one patient with missing restraint time documentation

^***^ Missing for the entire ED encounter in *n* = 117, pre-DEX in *n* = 254, post-DEX in *n* = 166

^****^ Missing for the entire ED encounter in *n* = 26, pre-DEX in *n* = 53, post-DEX in *n* = 224

Data on hemodynamics and vasopressors are presented in Table 3. There was no substantial change in nadir heart rate or mean arterial pressure after the initiation of DEX (median change in nadir heart rate 8 beats per minute [IQR −2, 23], median change in nadir mean arterial pressure 7 mm of mercury [IQR −8, 23]). There were small numerical increases in the use and dose of vasopressors from the pre-DEX phase to the post-DEX phase, but this was inconsistent across toxicologic condition categories.

**Table 3:** Hemodynamics and vasopressors

	EWD (*n* = 64)	AP-S (*n* = 63)	AP-M (*n* = 55)	AP-E (*n* = 52)	Others (*n* = 86)	Full Cohort (*n* = 320)
Nadir HR, bpm, median (IQR)	81.0 (71.0, 96.0)	71.5 (58.3, 83.8)	75.0 (66.8, 84.8)	69.5 (61.0, 82.0)	75.0 (62.8, 89.3)	74.0 (64.0, 88.0)
Pre-DEX	97.0 (80.5, 110)	88.0 (73.5, 111)	87.0 (72.0, 99.0)	83.5 (68.0, 93.0)	89.0 (74.3, 103)	88.0 (73.0, 105)
Missing, n (%)	1 (1.6%)	0 (0%)	2 (3.6%)	0 (0%)	0 (0%)	3 (0.9%)
Post-DEX	86.0 (73.8, 103)	74.0 (63.0, 88.0)	75.5 (67.0, 88.5)	73.5 (63.0, 83.5)	77.0 (64.0, 94.0)	76.0 (65.0, 93.3)
Missing, n (%)	12 (18.8%)	5 (7.9%)	1 (1.8%)	0 (0%)	2 (2.3%)	20 (6.3%)
Nadir HR < 60 bpm, *n* (%)	6 (9.4%)	17 (27.0%)	10 (18.2%)	10 (19.2%)	16 (18.6%)	59 (18.4%)
Pre-DEX	1 (1.6%)	8 (12.7%)	4 (7.3%)	6 (11.5%)	5 (5.8%)	24 (7.5%)
Missing, *n* (%)	1 (1.6%)	0 (0%)	2 (3.6%)	0 (0%)	0 (0%)	3 (0.9%)
Post-DEX	5 (7.8%)	11 (17.5%)	8 (14.5%)	9 (17.3%)	15 (17.4%)	48 (15.0%)
Missing, *n* (%)	12 (18.8%)	5 (7.9%)	1 (1.8%)	0 (0%)	2 (2.3%)	20 (6.3%)
Pre-post delta nadir HR, bpm, median (IQR)	7.00 (−7.00, 18.5)	9.50 (−2.50, 30.3)	6.50 (−0.250, 18.0)	5.50 (−2.00, 14.5)	10.0 (−2.00, 23.5)	8.00 (−2.00, 23.0)
Missing, *n* (%)	13 (20.3%)	5 (7.9%)	3 (5.5%)	0 (0%)	2 (2.3%)	23 (7.2%)
Nadir MAP, mmHg, median (IQR)	76.0 (67.0, 90.0)	77.0 (66.0, 87.0)	70.0 (62.5, 81.5)	66.0 (52.8, 73.5)	73.0 (55.3, 85.8)	72.0 (60.0, 85.0)
Pre-DEX	89.0 (76.0, 102)	89.0 (80.0, 101)	84.0 (71.0, 99.0)	75.5 (65.5, 86.5)	86.0 (74.0, 104)	85.0 (72.0, 100)
Missing, *n* (%)	1 (1.6%)	0 (0%)	2 (3.6%)	0 (0%)	1 (1.2%)	4 (1.3%)
Post-DEX	82.5 (67.0, 95.0)	80.0 (70.0, 97.0)	76.0 (66.0, 82.0)	70.5 (55.8, 78.8)	78.0 (61.3, 89.8)	76.0 (64.0, 90.0)
Missing, *n* (%)	14 (21.9%)	6 (9.5%)	2 (3.6%)	0 (0%)	4 (4.7%)	26 (8.1%)
Nadir MAP < 60 mmHg, *n* (%)	8 (12.5%)	6 (9.5%)	11 (20.0%)	20 (38.5%)	25 (29.1%)	70 (21.9%)
Pre-DEX	3 (4.7%)	3 (4.8%)	9 (16.4%)	9 (17.3%)	10 (11.6%)	34 (10.6%)
Missing, *n* (%)	1 (1.6%)	0 (0%)	2 (3.6%)	0 (0%)	1 (1.2%)	4 (1.3%)
Post-DEX	7 (10.9%)	5 (7.9%)	4 (7.3%)	17 (32.7%)	18 (20.9%)	51 (15.9%)
Missing, *n* (%)	14 (21.9%)	6 (9.5%)	2 (3.6%)	0 (0%)	4 (4.7%)	26 (8.1%)
Pre-post delta nadir MAP, mmHg, median (IQR)	2.00 (−7.00, 21.0)	8.00 (−1.00, 23.0)	6.00 (−11.0, 21.5)	5.50 (−12.3, 22.0)	8.00 (−4.75, 27.3)	7.00 (−7.50, 23.0)
Missing, *n* (%)	15 (23.4%)	6 (9.5%)	4 (7.3%)	0 (0%)	4 (4.7%)	29 (9.1%)
Any ED intravenous fluid resuscitation, *n* (%)	58 (90.6%)	41 (65.1%)	47 (85.5%)	42 (80.8%)	69 (80.2%)	257 (80.3%)
Pre-DEX	56 (87.5%)	33 (52.4%)	38 (69.1%)	29 (55.8%)	50 (58.1%)	206 (64.4%)
Post-DEX	35 (54.7%)	24 (38.1%)	30 (54.5%)	28 (53.8%)	43 (50.0%)	160 (50.0%)
Any vasopressor, *n* (%)	2 (3.1%)	10 (15.9%)	11 (20.0%)	12 (23.1%)	20 (23.3%)	55 (17.2%)
Pre-DEX	0 (0%)	5 (7.9%)	7 (12.7%)	2 (3.8%)	7 (8.1%)	21 (6.6%)
Post-DEX	2 (3.1%)	6 (9.5%)	4 (7.3%)	11 (21.2%)	15 (17.4%)	38 (11.9%)
Any infusion vasopressor, *n* (%)	2 (3.1%)	8 (12.7%)	9 (16.4%)	10 (19.2%)	19 (22.1%)	48 (15.0%)
Pre-DEX	0 (0%)	4 (6.3%)	6 (10.9%)	2 (3.8%)	6 (7.0%)	18 (5.6%)
Post-DEX	2 (3.1%)	5 (7.9%)	3 (5.5%)	8 (15.4%)	13 (15.1%)	31 (9.7%)
Maximum NEE infusion rate, mcg/kg/min, median (IQR)*	0.0900 (0.0850, 0.0950)	0.0500 (0.0390, 0.0500)	0.0700 (0.0500, 0.200)	0.0600 (0.0500, 0.0990)	0.150 (0.100, 0.325)	0.0800 (0.0500, 0.185)
Pre-DEX	-	0.0500 (0.0450, 0.0525)	0.0500 (0.0500, 0.163)	0.0390 (0.0345, 0.0435)	0.0950 (0.0350, 0.260)	0.0500 (0.0485, 0.120)
Post-DEX	0.0900 (0.0850, 0.0950)	0.0400 (0.0360, 0.0500)	0.0700 (0.0600, 0.135)	0.0550 (0.0500, 0.0840)	0.150 (0.100, 0.350)	0.0800 (0.0500, 0.175)
Any bolus vasopressor, *n* (%)	2 (3.1%)	5 (7.9%)	3 (5.5%)	6 (11.5%)	10 (11.6%)	26 (8.1%)
Pre-DEX	0 (0%)	4 (6.3%)	2 (3.6%)	1 (1.9%)	3 (3.5%)	10 (3.1%)
Post-DEX	2 (3.1%)	1 (1.6%)	1 (1.8%)	6 (11.5%)	8 (9.3%)	18 (5.6%)

Pre-post delta is the difference between the pre-DEX nadir vital sign and the post-DEX nadir vital sign rate. *EWD* Ethanol withdrawal, *AP-S* Acute poisoning by sympathomimetics, *AP-M* Acute poisoning by multiple substance classes, *AP-E* Acute poisoning by ethanol, *DEX* Dexmedetomidine, *HR* Heart rate, *bpm* Beats per minutes, *IQR* Interquartile range, *MAP* Mean arterial pressure, *mmHg* millimeters of mercury, *NEE* Norepinephrine equivalent

^*^Includes only patients receiving any infusion vasopressors during the specified period

One hundred twenty patients (37.5%) were intubated in the ED *prior to the initiation of DEX*. Of the remaining 200 patients, 43 (21.5% of those at risk) were intubated after DEX initiation, and 157 (78.5% of those at risk) were not, for an intubation frequency of 21.5% (95% CI 16–28%). Data segregated by intubation status are presented in Table 4.

**Table 4:** Intubation status

	Intubated Pre-DEX (*n* = 120)	Intubated Post-DEX (*n* = 43)	Not Intubated Post-DEX (*n* = 157)
Candidate Factors for Multivariable Model			
Blood ethanol, mg/dL, median (IQR)*	23.5 (0, 237)	0 (0, 0)	0 (0, 122)
Blood ethanol not tested, *n* (%)	24 (20%)	14 (32.6%)	64 (40.8%)
Blood ethanol detectable, *n* (%)	51 (42.5%)	7 (16.3%)	39 (24.8%)
Blood ethanol undetectable, *n* (%)	45 (37.5%)	22 (51.2%)	54 (34.4%)
Any benzodiazepine prior to intubation, *n* (%)	35 (29.2%)	38 (88.4%)	116 (73.9%)
Any antipsychotic prior to intubation, *n* (%)	24 (20%)	32 (74.4%)	82 (52.2%)
Any ketamine prior to intubation, *n* (%)	8 (6.7%)	10 (23.3%)	35 (22.3%)
Other Variables (Descriptive)			
Study site, *n* (%)			
BJH	86 (71.7%)	22 (51.2%)	104 (66.2%)
HCMC	34 (28.3%)	21 (48.8%)	53 (33.8%)
Tox condition, *n* (%)			
Withdrawal, ethanol	15 (12.5%)	8 (18.6%)	41 (26.1%)
Acute poisoning, sympathomimetics	18 (15.0%)	11 (25.6%)	34 (21.7%)
Acute poisoning, multiple classes	26 (21.7%)	3 (7.0%)	26 (16.6%)
Acute poisoning, ethanol	28 (23.3%)	4 (9.3%)	20 (12.7%)
Other conditions	33 (27.5%)	17 (39.5%)	36 (22.9%)
Pre-DEX RASS, median (IQR)	−1 (−2, 1)	4 (4, 4)	0 (−1, 1)
Missing, *n* (%)	63 (52.5%)	42 (97.7%)	149 (94.9%)
Pre-DEX GCS, median (IQR)	7 (3, 13)	14 (13, 15)	14 (12, 15)
Missing, *n* (%)	20 (16.7%)	6 (14.0%)	27 (17.2%)
Any ED vasopressor, *n* (%)	35 (29.2%)	9 (20.9%)	11 (7.0%)
Any ED infusion vasopressor, *n* (%)	31 (25.8%)	7 (16.3%)	10 (6.4%)
Maximum norepinephrine equivalent rate, mcg/kg/min, median (IQR)	0.07 (0.05, 0.13)	0.3 (0.1, 0.82)	0.08 (0.06, 0.17)
Any ED bolus vasopressor, *n* (%)	14 (11.7%)	6 (14.0%)	6 (3.8%)
Any ED NPPV, n (%)	14 (11.7%)	12 (27.9%)	42 (26.8%)
Any IV fluid resuscitation, *n* (%)	104 (86.7%)	32 (74.4%)	121 (77.1%)
ED disposition, *n* (%)			
ICU	106 (88.3%)	42 (97.7%)	127 (80.9%)
Stepdown/OU	1 (0.8%)	0	2 (1.3%)
Floor	5 (4.2%)	0	9 (5.7%)
Discharged	8 (6.7%)	1 (2.3%)	18 (11.5%)
Expired	0	0	1 (0.6%)
Hospital mortality, *n* (%)	3 (2.5%)	1 (2.3%)	4 (2.5%)

*DEX* Dexmedetomidine, *IQR* = Interquartile range, BJH = Barnes-Jewish Hospital, HCMC = Hennepin County Medical Center, ED = emergency department, *NPPV* Non-invasive positive pressure ventilation

^*^Excludes patients not tested for ethanol

On our primary analysis, the administration of antipsychotics to patients at risk for intubation was associated with an increased odds of intubation (adjusted OR 2.48, 95% CI 1.17–5.57). Our first sensitivity analysis, incorporating a random effect for study site, yielded an adjusted ICC of 0.024, suggesting that only 2.4% of variance was attributable to between-site differences, and the findings did not differ substantially from the primary model.

In our second sensitivity analysis, which treated blood ethanol concentration as a continuous variable and dropped patients who were not tested, none of the tested factors were significantly associated with intubation. In our third sensitivity analysis, which extended the definition of intubation to include the full 24 h after DEX initiation (see Appendix 6), none of the tested factors were significantly associated with intubation Comprehensive details of the statistical analyses and models are presented in Appendix 7.

## Discussion

In this dual-center retrospective cohort study of the use of DEX for toxicologic conditions, we found that DEX was used primarily to treat patients with ethanol withdrawal, sympathomimetic intoxication, acute poisoning by multiple xenobiotic classes, and ethanol intoxication. Co-administration of other sedatives was universal. Bradycardia and hypotension did occur, and while there was no substantial change in nadir heart rate or mean arterial pressure after DEX initiation, there was an increase in the use and dose of vasopressors after DEX initation (approximately 6.6% to 11.9%). Approximately a fifth of patients who were not already intubated when DEX was initiated were intubated after DEX initiation, and this outcome appeared to be associated with co-administration of antipsychotics.

In our study, the primary toxicologic conditions were ethanol withdrawal, sympathomimetic poisoning (predominantly amphetamines and cocaine) multiple substance poisoning, and ethanol intoxication. In contrast, in a prior study of the use of DEX in patients with toxicologic conditions, Kershner and colleagues [10] found that antimuscarinics were the leading category (27%), although sedative withdrawal, sympathomimetics, and opioid withdrawal were also represented. In another prior study by Mohorn and colleagues [9], seven cases (32%) involved ethanol or agents potentially consistent with drugs of misuse. These differences are likely explained by our differing populations (Kershner and colleagues included only patients who were not intubated when DEX was started, and Mohorn and colleagues included only intubated patients), sample sizes, and time periods. Our findings in this study are, however, consistent with our previous systematic review of DEX for toxicologic conditions, in which stimulant poisoning and sedative withdrawal were the most commonly identified indications[8]. Notably, in our study, HCMC used DEX substantially more frequently in patients with ethanol as the culprit xenobiotic; we suspect that this is due to hospital-level variations in the use of DEX for alcohol withdrawal, a topic of continuing clinical controversy [25–27]. Regional differences in substance use disorder patterns may also explain site differences. HCMC has a dedicated, well-staffed ED unit for the care of intoxicated patients and cares for a large number of alcohol-related visits [28]. HCMC is also located in a region of the U.S. in which binge drinking is more prevalent [29, 30].

We found a relatively high frequency of intubation following DEX initiation. In contrast, Kershner and colleagues reported that only four patients (6%) of their 70-patient cohort were ultimately intubated after DEX initiation; three of these patients had received benzodiazepines as well [10]. This difference is likely explained by our populations. Kershner and colleagues identified their cohort via calls to a poison center and included patients started on DEX later in their clinical course. We included all patients seen at our study EDs who had *any* toxicologic condition, whether or not it was the sole or primary driver of the patient's presentation, and may have identified many patients with other non-toxicologic and non-DEX indications for intubation. The relatively high utilization of vasopressors and non-invasive positive pressure ventilation in our cohort, as well as the low but non-zero mortality, also suggest that we identified a generally sicker cohort than Kershner and colleagues. Our finding that antipsychotic administration was associated with an increased odds of intubation was unexpected, as we typically do not expect therapeutic antipsychotics to depress the respiratory drive [31]. Antipsychotic use may be a blunt proxy for more severe agitation or more severe withdrawal. We also note that this finding did not persist in our sensitivity analyses. This association should be investigated in future studies.

DEX is well-known to cause bradycardia, and to a lesser extent hypotension [1]; this has been demonstrated in patients receiving DEX to facilitate non-invasive positive pressure ventilation[3] and mechanical ventilation [32, 33], as well as our previous prospective study of DEX use for any indication in the ED [24]. However, it is not clear that DEX-induced bradycardia or mild DEX-induced hypotension are clinically relevant or usually lead to any intervention [6, 23]. In our study, the median *nadir* heart rate and mean arterial pressure were in the normal range after DEX initiation, and there was no substantial change in these vital signs from pre- to post-DEX, although a small proportion of patients did have some bradycardia or hypotension after DEX initiation. We did, however, note small but non-zero increases in the use and rate of vasopressors after DEX initiation, although we are unable to determine whether this was related to DEX or the underlying condition being treated. Mohorn and colleagues reported that 23% of their patients experienced bradycardia or hypotension, but used a relatively strict definition (greater than 30% change in baseline heart rate or blood pressure), and reported absent or clinically modest changes in blood pressure (median systolic blood pressure 119 mm of mercury pre-DEX and 111 post-DEX, *p* = 0.745) and heart rate (median heart rate 93 beats per minute pre-DEX and 82 post-DEX, *p* < 0.05) after DEX initiation in their total study sample [9]. Kershner and colleagues also found modest changes (median mean arterial pressure 99 mm of mercury pre-DEX and 87 post-DEX; median heart rate 107 beats per minute pre-DEX and 84 post-DEX), and reported that bradycardia and hypotension each occurred in only one patient (1%) [10]. Taken together, these findings indicate that DEX at the relatively low infusion rates used in non-intubated patients does not generally appear to induce clinically significant reductions in heart rate or blood pressure in patients with toxicologic conditions, but that a small subset of patients may experience bradycardia or hypotension.

It is challenging to comment on the clinical effectiveness of DEX in the context of our study. We attempted to evaluate sedation depth before and after DEX, but this analysis was limited by the high missingness of RASS and GCS data. While we observed tendencies towards reduction in the administration of other sedatives (see Appendix 5), we did not have the statistical power to perform formal hypothesis testing for these secondary outcomes, and it is possible that this effect simply reflects the relative amount of time in the ED before and after DEX initiation. In future work, we hope to obtain regular assessments of sedation depth and time-adjust our sedative data.

Our study has several important limitations. First, this was a retrospective chart review study and suffered from the usual limitations of this study design, including inconsistent and non-standardized documentation of variables of interest. Second, we did not require that included patients had *exclusively* toxicologic conditions; that is, other serious acute medical and surgical conditions such as trauma and sepsis were likely also present in our sample, which may explain the relatively high frequencies of interventions not usually associated with the toxicologic conditions we identified. Although this limits the internal validity of our work, it enhances its generalizability and pragmatism, as the cohort we identified is more representative of usual medical practice than an artificially constrained sample of patients with exclusively toxicologic conditions. Third, we did not conduct dual adjudication of toxicologic conditions or dual blinded data abstraction. However, determination of the presence of a toxicologic condition by a single toxicologist is usual practice in the ToxIC registry [16], and we assessed only variables and outcomes that were objectively present in the EMR as discrete data, which limits subjectivity and reduces the risk of substantial variance between abstractors. Fourth, we are unable to determine if patients were intubated after DEX initiation because of inadequate effects of DEX, adverse effects of DEX, or some entirely unrelated factor, and we were unable to account for clustering by individual clinicians' practice patterns or formally analyze variations across toxicologic condition categories due to our small sample size. Finally, our study was descriptive and hypothesis-generating and had a relatively small sample size. Accordingly, clinical factors that were not statistically significant should be interpreted cautiously, given the limited statistical power and exploratory and hypothesis-generating nature of the analysis, the high degree of missing data on pre-DEX agitation and sedation scores, and the intrinsically multifactorial and clinically nuanced nature of the decision to intubate.

Future research in this area should directly compare DEX to other treatments and should be conducted prospectively. Future studies should integrate evaluation not only of patients but of the clinicians caring for them, in order to capture data on what factors drive decision-making regarding DEX use, intubation, and other treatments. Larger sample sizes, which will likely require multicenter collaborations, will increase statistical power and more clearly describe existing practice pattern variation. Future investigators should consider assessing and controlling for co-existing non-toxicologic conditions and limiting their investigations to a single relatively common toxicologic condition or stratifying by condition. Ultimately, interventional or pseudo-interventional studies may be necessary to determine the true effect of DEX in the treatment of common toxicologic conditions. For ethical, regulatory, and logistical reasons, these will likely need to employ techniques other than patient-level randomization, such as cluster randomization by hospital [34], target trial emulation [35], or rigorous pre-post evaluations [36].

## Conclusion

In this dual-center retrospective cohort study, DEX was primarily used to treat ED patients with intoxication or withdrawal from ethanol and substances of misuse. Clinically substantial changes in blood pressure and heart rate were not observed. Among patients who were not intubated when DEX was initiated, the frequency of intubation after DEX initiation was 21.5%. Co-administration of antipsychotics was associated with increased odds of intubation. Further prospective research is needed to evaluate DEX in the treatment of toxicologic conditions.

## Supplementary Information

Below is the link to the electronic supplementary material.

Supplementary file1 (DOC 84 KB)

Supplementary file2 (DOCX 15 KB)

Supplementary file3 (DOCX 17 KB)

Supplementary file4 (DOCX 18 KB)

Supplementary file5 (DOCX 48 KB)

Supplementary file6 (DOCX 21 KB)

Supplementary file7 (DOCX 34 KB)

## Data Availability

Preliminary data from this study were previously presented at the American College of Medical Toxicology Annual Scientific Meeting in Boston, MA, United States of America, in March 2026.
