# Supplementary material for: Dexmedetomidine in The Treatment of Toxicologic Conditions in The Emergency Department: A Dual-Center Retrospective Observational Cohort Study
Source: J Med Toxicol. 2026 Jul 10;22(3):364–74. doi: 10.1007/s13181-026-01145-5 (PMC13407800; doi:10.1007/s13181-026-01145-5)
Supplement: Supplementary file 2 — Supplementary file2 (DOCX 15 KB) [file 13181_2026_1145_MOESM2_ESM.docx]

**APPENDIX 2: Selected Additional Variable Definitions**

| **Variable** | **Definition** |
| --- | --- |
| Vasopressor | Any vasopressor or inotrope, administered as an intravenous continuous infusion or bolus, defined as any of the following agents: norepinephrine, epinephrine, phenylephrine, vasopressin, dopamine, dobutamine, angiotensin II, methylene blue (infusion only). |
| Infusion vasopressor | Any vasopressor or inotrope administered as an intravenous continuous infusion, defined as any of the following agents: norepinephrine, epinephrine, phenylephrine, vasopressin, dopamine, dobutamine, angiotensin II. |
| Bolus vasopressor | Any vasopressor or inotrope administered as an intravenous bolus, defined as epinephrine or phenylephrine . |
| Intravenous fluid resuscitation | Administration of a crystalloid fluid as an intravenous bolus. Excludes crystalloids administered by continuous infusion, colloids, and dextrose-containing fluids. |
| Non-invasive positive pressure ventilation | Delivery of respiratory support via bilevel positive airway pressure (BiPAP), continuous positive airway pressure (CPAP), or high-flow nasal cannula. |
| Primary culprit xenobiotic class and agent | The class and agent deemed primarily responsible for the patient's presentation by the reviewing attending medical toxicologist. |
| Restraint | Application of soft-limb restraints, hard or locked-limb restraints, or mittens as restraint. |

**Supplementary Table 1. Selected additional variable definitions.**
