# Supplementary material for: Dexmedetomidine in The Treatment of Toxicologic Conditions in The Emergency Department: A Dual-Center Retrospective Observational Cohort Study
Source: J Med Toxicol. 2026 Jul 10;22(3):364–74. doi: 10.1007/s13181-026-01145-5 (PMC13407800; doi:10.1007/s13181-026-01145-5)
Supplement: Supplementary file 3 — Supplementary file3 (DOCX 17 KB) [file 13181_2026_1145_MOESM3_ESM.docx]

|  | **BJH**  **(n=212)** | **HCMC**  **(n=108)** | **Full Cohort (n=320)** |
| --- | --- | --- | --- |
| **Toxicologic condition, n (%)** |  |  |  |
| Withdrawal, ethanol | 11 (5.2%) | 53 (49.1%) | 64 (20%) |
| Acute poisoning, sympathomimetics | 54 (25.5%) | 9 (8.3%) | 63 (19.7%) |
| Acute poisoning, multiple classes | 34 (16.0%) | 21 (19.4%) | 55 (17.2%) |
| Acute poisoning, ethanol | 42 (19.8%) | 10 (9.3%) | 52 (16.3%) |
| Other conditions | 71 (33.5%) | 15 (13.9%) | 86 (26.9%) |
| Acute poisoning, other single class | 50 (23.6%) | 3 (2.8%) | 53 (16.6%) |
| Withdrawal, other | 10 (4.7%) | 1 (0.9%) | 14 (4.4%) |
| Adverse drug event | 3 (1.4%) | 5 (4.6%) | 11 (3.4% |
| Mixed presentation | 8 (3.8%) | 6 (5.6%) | 8 (2.5%) |

**Supplementary Table S1. Toxicologic conditions by study site.** * Missing data for one patient whose identity was never determined. ** Missing data for 7 patients due to missing weight or height in the ED record. *** BJH = Barnes-Jewish Hospital, HCMC = Hennepin County Medical Center.
