# Supplementary material for: Dexmedetomidine in The Treatment of Toxicologic Conditions in The Emergency Department: A Dual-Center Retrospective Observational Cohort Study
Source: J Med Toxicol. 2026 Jul 10;22(3):364–74. doi: 10.1007/s13181-026-01145-5 (PMC13407800; doi:10.1007/s13181-026-01145-5)
Supplement: Supplementary file 4 — Supplementary file4 (DOCX 18 KB) [file 13181_2026_1145_MOESM4_ESM.docx]

**APPENDIX 4: TOXICOLOGIC CONDITION AND XENOBIOTIC CLASS DATA**

| **Condition** | **N** |
| --- | --- |
| **Intoxication or acute poisoning** | 223 |
| *Single primary agent* | 168 |
| Alcohol (ethanol) | 52 |
| Antidepressant | 2 |
| Antipsychotic | 1 |
| Caustic | 1 |
| Gas, vapor, dust, or irritant | 2 |
| Lithium | 2 |
| Opioid | 41 |
| Psychoactive | 2 |
| Sedative-hypnotic | 1 |
| Sympathomimetic | 63 |
| Unknown | 1 |
| *Multiple primary agents** | 55 |
| Alcohol (ethanol) | 13 |
| Anticholinergic/antihistamine | 2 |
| Antidepressant | 3 |
| Antipsychotic | 3 |
| Gas, vapor, dust, or irritant | 1 |
| Opioid | 32 |
| Psychoactive | 11 |
| Sedative-hypnotic | 8 |
| Sympathomimetic | 36 |
| **Withdrawal syndrome** | 78 |
| *Single primary agent* | 76 |
| Alcohol (ethanol) | 64 |
| Opioid | 7 |
| Sedative-hypnotic | 4 |
| Sympathomimetic | 1 |
| *Multiple primary agents** | 2 |
| Opioid | 2 |
| Sedative-hypnotic | 1 |
| Sympathomimetic | 1 |
| **Adverse drug event** | 11 |
| *Single primary agent* | 10 |
| Alcohol (ethanol) | 1 |
| Antidepressant | 1 |
| Cardiovascular | 3 |
| Opioid | 1 |
| Other non-pharmaceutical | 1 |
| Other pharmaceutical | 2 |
| Parkinsons | 1 |
| *Multiple primary agents** | 1 |
| Anticholinergic/antihistamine | 1 |
| Sedative-hypnotic | 1 |
| **Mixed** | 8 |
| *Single primary agent* | 1 |
| Alcohol (ethanol) | 1 |
| *Multiple primary agents** | 7 |
| Alcohol (ethanol) | 1 |
| Antipsychotic | 1 |
| Diabetes | 1 |
| Opioid | 6 |
| Sympathomimetic | 5 |

**Supplementary Table S3. Toxicologic conditions and xenobiotic classes.** * Number for multi-primary cases represent the number of cases *including* the given xenobiotic class. These numbers will sum to greater than the total number of cases. ** All intoxication/acute poisoning plus withdrawal, except for one case of adverse drug event plus withdrawal.
