# Supplementary material for: Dexmedetomidine in The Treatment of Toxicologic Conditions in The Emergency Department: A Dual-Center Retrospective Observational Cohort Study
Source: J Med Toxicol. 2026 Jul 10;22(3):364–74. doi: 10.1007/s13181-026-01145-5 (PMC13407800; doi:10.1007/s13181-026-01145-5)
Supplement: Supplementary file 5 — Supplementary file5 (DOCX 48 KB) [file 13181_2026_1145_MOESM5_ESM.docx]

|  | **EWD (n=64)** | **AP-S (n=63)** | **AP-M (n=55)** | **AP-E (n=52)** | **Others (n=86)** | **Full Cohort (n=320)** |
| --- | --- | --- | --- | --- | --- | --- |
| **Toxidrome, n (%)** |  |  |  |  |  |  |
| Sympathomimetic | 1 (1.6%) | 53 (84.1%) | 21 (38.2%) | 1 (1.9%) | 4 (4.7%) | 80 (25%) |
| Opioid | 0 (0%) | 9 (14.3%) | 16 (29.1%) | 0 (0%) | 43 (50%) | 68 (21.3%) |
| Sedative-hypnotic | 1 (1.6%) | 0 (0%) | 8 (14.5%) | 38 (73.1%) | 5 (5.8%) | 52 (16.3%) |
| Alcoholic ketoacidosis | 6 (9.4%) | 0 (0%) | 0 (0%) | 1 (1.9%) | 1 (1.2%) | 8 (2.5%) |
| Anticholinergic | 0 (0%) | 0 (0%) | 1 (1.8%) | 0 (0%) | 1 (1.2%) | 2 (0.6%) |
| Serotonin Syndrome | 0 (0%) | 0 (0%) | 0 (0%) | 1 (1.9%) | 1 (1.2%) | 2 (0.6%) |
| Sympatholytic | 0 (0%) | 0 (0%) | 1 (1.8%) | 0 (0%) | 0 (0%) | 1 (0.3%) |
| **Any gastrointestinal decontamination, n (%)** | 0 (0%) | 0 (0%) | 1 (1.8%) | 0 (0%) | 0 (0%) | 1 (0.3%) |
| **Maximum ED QRS duration, ms, median (IQR)*** | 92 (86, 101) | 90 (84, 98) | 96 (86, 102) | 94 (86.5, 101) | 90 (82, 100) | 92 (84, 100) |
| **Maximum ED QTc duration, ms, median (IQR)*** | 409 (388, 443) | 456 (429, 471) | 449 (426, 478) | 458 (425, 487) | 458 (436, 478) | 450 (414, 473) |
| **At risk of dysrhythmia by QT nomogram, n (%)*** | 16 (25.0%) | 4 (6.3%) | 10 (18.2%) | 6 (11.5%) | 13 (15.1%) | 49 (15.3%) |
| **Benzodiazepines** |  |  |  |  |  |  |
| Total milligram lorazepam equivalents,  median (IQR) |  |  |  |  |  |  |
| ED encounter | 218 (42.6, 652) | 20 (7.75, 30.5) | 15 (4.5, 33.4) | 8 (4, 22.5) | 7.7 (4, 42.5) | 20 (6, 102) |
| Pre-DEX | 214 (21.3, 500) | 11 (4, 28.5) | 9 (2.25, 19.5) | 6 (1.75, 12) | 5.04 (2, 19) | 11 (4, 59) |
| Post-DEX | 0 (0, 7.5) | 0 (0, 8.5) | 0.25 (0, 8) | 2 (0, 5.68) | 1 (0, 4) | 0 (0, 8) |
| Lorazepam, n (%) |  |  |  |  |  |  |
| ED encounter | 33 (51.6%) | 19 (30.2%) | 19 (34.5%) | 10 (19.2%) | 32 (37.2%) | 113 (35.3%) |
| Pre-DEX | 32 (50%) | 17 (27%) | 17 (30.9%) | 8 (15.4%) | 27 (31.4%) | 101 (31.6%) |
| Post-DEX | 3 (4.7%) | 5 (7.9%) | 6 (10.9%) | 4 (7.7%) | 11 (12.8%) | 29 (9.1%) |
| Lorazepam dose, mg, median (IQR) |  |  |  |  |  |  |
| ED encounter | 6 (2, 8) | 6 (3.5, 8) | 3 (2, 6) | 2 (1.25, 5) | 2.5 (2, 6) | 4 (2, 8) |
| Pre-DEX | 6 (2, 8) | 5 (2, 8) | 3 (1.5, 5) | 1.5 (1, 5) | 2 (1, 4.25) | 3 (2, 7) |
| Post-DEX | 0 (0, 0) | 0 (0, 2) | 0 (0, 0.625) | 0 (0, 2) | 0 (0, 1) | 0 (0, 0.5) |
| Diazepam, n (%) |  |  |  |  |  |  |
| ED encounter | 47 (73.4%) | 8 (12.7%) | 7 (12.7%) | 2 (3.8%) | 10 (11.6%) | 74 (23.1%) |
| Pre-DEX | 45 (70.3%) | 7 (11.1%) | 5 (9.1%) | 2 (3.8%) | 7 (8.1%) | 66 (20.6%) |
| Post-DEX | 12 (18.8%) | 2 (3.2%) | 4 (7.3%) | 0 (0%) | 5 (5.8%) | 23 (7.2%) |
| Diazepam dose, mg, median (IQR) |  |  |  |  |  |  |
| ED encounter | 80 (35, 145) | 12.5 (10, 22.5) | 20 (7.5, 25) | 80 (45, 115) | 17.5 (10, 27.5) | 40 (20, 120) |
| Pre-DEX | 70 (35, 125) | 10 (9.38, 12.5) | 10 (2.5, 20) | 80 (45, 115) | 10 (2.5, 17.5) | 40 (10, 97.5) |
| Post-DEX | 0 (0, 5) | 0 (0, 1.25) | 5 (0, 10) | 0 (0, 0) | 2.5 (0, 17.5) | 0 (0, 10) |
| Any midazolam, n (%) |  |  |  |  |  |  |
| ED encounter | 14 (21.9%) | 26 (41.3%) | 31 (56.4%) | 20 (38.5%) | 26 (30.2%) | 117 (36.6%) |
| Pre-DEX | 12 (18.8%) | 23 (36.5%) | 27 (49.1%) | 17 (32.7%) | 20 (23.3%) | 99 (30.9%) |
| Post-DEX | 6 (9.4%) | 12 (19%) | 12 (21.8%) | 12 (23.1%) | 15 (17.4%) | 57 (17.8%) |
| Bolus-dose midazolam, n (%) |  |  |  |  |  |  |
| ED encounter | 13 (20.3%) | 24 (38.1%) | 23 (41.8%) | 19 (36.5%) | 24 (27.9%) | 103 (32.2%) |
| Pre-DEX | 10 (15.6%) | 21 (33.3%) | 18 (32.7%) | 16 (30.8%) | 17 (19.8%) | 82 (25.6%) |
| Post-DEX | 6 (9.4%) | 12 (19.0%) | 11 (20.0%) | 11 (21.2%) | 13 (15.1%) | 53 (16.6%) |
| Bolus-dose midazolam dose, mg, median (IQR) |  |  |  |  |  |  |
| ED encounter | 50 (4, 8) | 8.5 (5, 11.3) | 5 (2, 10) | 5 (2, 10) | 4 (2, 10) | 5 (2, 10) |
| Pre-DEX | 4 (2, 6) | 5 (2, 6.75) | 4 (2, 6) | 4 (1.5, 5) | 4 (0, 4.25) | 4 (2, 6) |
| Post-DEX | 0 (0, 3) | 1 (0, 4.25) | 0 (0, 3) | 2 (0, 5) | 1 (0, 3.5) | 1 (0, 4) |
| Midazolam infusion, n (%) |  |  |  |  |  |  |
| ED encounter | 1 (1.6%) | 2 (3.2%) | 3 (5.5%) | 1 (1.9%) | 3 (3.5%) | 10 (3.1%) |
| Pre-DEX | 1 (1.6%) | 0 (0%) | 1 (1.8%) | 1 (1.9%) | 2 (2.3%) | 5 (1.6%) |
| Post-DEX | 1 (1.6%) | 2 (3.2%) | 3 (5.5%) | 1 (1.9%) | 3 (3.5%) | 10 (3.1%) |
| Time-weighted average midazolam infusion rate, mg/h, median (IQR) |  |  |  |  |  |  |
| ED encounter | 4.67 (4.67, 4.67) | 5.66 (3.49, 7.84) | 1.22 (0.9, 1.60) | 1.70 (1.7, 1.7) | 3.13 (2.06, 3.55) | 1.84 (1.24, 3.76) |
| Pre-DEX | 1.11 (1.11, 1.11) | 0 (0, 0) | 0 (0, 0.500) | 1.49 (1.49, 1.49) | 2.86 (1.43, 3.38) | 0.5 (0, 1.4) |
| Post-DEX | 4.76 (4.76, 4.76) | 5.66 (3.49, 7.84) | 1.24 (0.91, 1.61) | 2.45 (2.45, 2.45) | 3.81 (2.4, 3.9) | 2.21 (1.26, 3.95) |
| **Antipsychotics** |  |  |  |  |  |  |
| Droperidol, n (%) |  |  |  |  |  |  |
| ED encounter | 6 (9.4%) | 18 (28.6%) | 17 (30.9%) | 6 (11.5%) | 15 (17.4%) | 62 (19.4%) |
| Pre-DEX | 5 (7.8%) | 17 (27%) | 16 (29.1%) | 6 (11.5%) | 11 (12.8%) | 55 (17.2%) |
| Post-DEX | 1 (1.6%) | 1 (1.6%) | 5 (9.1%) | 2 (3.8%) | 5 (5.8%) | 14 (4.4%) |
| Droperidol dose, mg, median (IQR) |  |  |  |  |  |  |
| ED encounter | 2.5 (2.5, 8.13) | 5 (2.81, 8.75) | 10 (5, 20) | 6.88 (5.31, 9.38) | 5 (2.5, 10) | 5 (2.5, 10) |
| Pre-DEX | 2.5 (2.5, 8.13) | 5 (2.5, 8.75) | 10 (5, 15) | 5.63 (5, 9.06) | 2.5 (0.313, 8.75) | 5 (2.5, 10) |
| Post-DEX | 0 (0, 0) | 0 (0, 0) | 0 (0, 2.5) | 0 (0, 1.88) | 0 (0, 2.5) | 0 (0, 0) |
| Haloperidol, n (%) |  |  |  |  |  |  |
| ED encounter | 5 (7.8%) | 16 (25.4%) | 13 (23.6%) | 12 (23.1%) | 13 (15.1%) | 59 (18.4%) |
| Pre-DEX | 4 (6.3%) | 12 (19%) | 9 (16.4%) | 10 (19.2%) | 11 (12.8%) | 46 (14.4%) |
| Post-DEX | 1 (1.6%) | 6 (9.5%) | 3 (5.5%) | 2 (3.8%) | 3 (3.5%) | 15 (4.7%) |
| Haloperidol dose, mg, median (IQR) |  |  |  |  |  |  |
| ED encounter | 10 (10, 15) | 10 (5, 20) | 10 (5, 10) | 10 (5, 10) | 10 (5, 10) | 10 (5, 12.5) |
| Pre-DEX | 10 (5, 10) | 7.5 (3, 15) | 10 (0, 10) | 9 (5, 10) | 5 (5, 10) | 8 (4.5, 10) |
| Post-DEX | 0 (0, 0) | 0 (0, 5) | 0 (0, 0) | 0 (0, 0) | 0 (0, 0) | 0 (0, 1) |
| Olanzapine, n (%) |  |  |  |  |  |  |
| ED encounter | 16 (25%) | 6 (9.5%) | 16 (29.1%) | 8 (15.4%) | 15 (17.4%) | 61 (19.1%) |
| Pre-DEX | 13 (20.3%) | 6 (9.5%) | 15 (27.3%) | 7 (13.5%) | 14 (16.3%) | 55 (17.2%) |
| Post-DEX | 3 (4.7%) | 1 (1.6%) | 1 (1.8%) | 2 (3.8%) | 1 (1.2%) | 8 (2.5%) |
| Olanzapine dose, mg, median (IQR) |  |  |  |  |  |  |
| ED encounter | 10 (5, 10) | 20 (20, 20) | 15 (10, 20) | 12.5 (8.75, 16.3) | 15 (10, 20) | 10 (10, 20) |
| Pre-DEX | 10 (4.38, 10) | 20 (16.3, 20) | 15 (10, 20) | 12.5 (5, 15) | 10 (10, 18.8) | 10 (10, 20) |
| Post-DEX | 0 (0, 0) | 0 (0, 0) | 0 (0, 0) | 0 (0, 2.5) | 0 (0, 0) | 0 (0, 0) |
| **Propofol** |  |  |  |  |  |  |
| Bolus-dose propofol, n (%) |  |  |  |  |  |  |
| ED encounter | 10 (15.6%) | 15 (23.8%) | 16 (29.1%) | 11 (21.2%) | 24 (27.9%) | 76 (23.8%) |
| Pre-DEX | 5 (7.8%) | 11 (17.5%) | 10 (18.2%) | 7 (13.5%) | 15 (17.4%) | 48 (15.0%) |
| Post-DEX | 6 (9.4%) | 7 (11.1%) | 9 (16.4%) | 4 (7.7%) | 12 (14.0%) | 38 (11.9%) |
| Bolus-dose propofol dose, mg, median (IQR) |  |  |  |  |  |  |
| ED encounter | 95 (47.5, 107) | 50 (35, 80) | 40 (33.8, 97.5) | 30 (20, 40) | 55 (50, 65) | 50 (30, 82.5) |
| Pre-DEX | 15 (0, 52.5) | 30 (5, 55) | 25 (0, 36.3) | 20 (0, 30) | 35 (0, 52.5) | 25 (0, 50) |
| Post-DEX | 35 (0, 85) | 0 (0, 35) | 20 (0, 42.5) | 0 (0, 15) | 10 (0, 52.5) | 50 (0, 41.3) |
| Propofol infusion, n (%) |  |  |  |  |  |  |
| ED encounter | 22 (34.4%) | 24 (38.1%) | 25 (45.5%) | 23 (44.2%) | 40 (46.5%) | 134 (41.9%) |
| Pre-DEX | 15 (23.4%) | 16 (25.4%) | 21 (38.2%) | 17 (32.7%) | 21 (24.4%) | 90 (28.1%) |
| Post-DEX | 22 (34.4%) | 22 (34.9%) | 23 (41.8%) | 21 (40.4%) | 37 (43.0%) | 125 (39.1%) |
| Time-weighted average propofol infusion rate, mg/h, median (IQR) |  |  |  |  |  |  |
| ED encounter | 52.4 (41.3, 66.8) | 35.4 (25.9, 40.2) | 35 (26.9, 49) | 32.4 (21.7, 39) | 36 (24.7, 44.1) | 36.3 (26.3, 46.7) |
| Pre-DEX | 42 (0, 67.9) | 24.8 (0, 35.5) | 35.6 (23.7, 44.9) | 21.1 (5.61, 39.6) | 9.85 (0, 32.2) | 27.8 (0, 40.9) |
| Post-DEX | 47.2 (41.3, 62.7) | 35.3 (24.3, 49.1) | 35.2 (20.9, 44.2) | 30.6 (19.5, 38.9) | 35.3 (22.3, 45.5) | 36.5 (21.9, 48.6) |
| **Ketamine** |  |  |  |  |  |  |
| Bolus-dose ketamine, n (%) |  |  |  |  |  |  |
| ED encounter | 1 (1.6%) | 20 (31.7%) | 17 (30.9%) | 13 (25.0%) | 26 (30.2%) | 77 (24.1%) |
| Pre-DEX | 1 (1.6%) | 17 (27%) | 14 (25.5%) | 8 (15.4%) | 19 (22.1%) | 59 (18.4%) |
| Post-DEX | 0 (0%) | 5 (7.9%) | 5 (9.1%) | 7 (13.5%) | 11 (12.8%) | 28 (8.8%) |
| Bolus-dose ketamine dose, mg, median (IQR) |  |  |  |  |  |  |
| ED encounter | 60 (60, 60) | 72.5 (20, 103) | 100 (50, 155) | 150 (50, 200) | 100 (100, 145) | 100 (50, 155) |
| Pre-DEX | 60 (60, 60) | 50 (20, 92.5) | 50 (20, 125) | 80 (0, 146) | 100 (2.5, 118) | 70 (10, 110) |
| Post-DEX | 0 (0, 0) | 0 (0, 2.5) | 0 (0, 60) | 40 (0, 50) | 0 (0, 47.5) | 0 (0, 50.) |
| Ketamine infusion, n (%) |  |  |  |  |  |  |
| ED encounter | 0 (0%) | 1 (1.6%) | 2 (3.6%) | 0 (0%) | 0 (0%) | 3 (0.9%) |
| Pre-DEX | 0 (0%) | 0 (0%) | 2 (3.6%) | 0 (0%) | 0 (0%) | 2 (0.6%) |
| Post-DEX | 0 (0%) | 1 (1.6%) | 2 (3.6%) | 0 (0%) | 0 (0%) | 3 (0.9%) |
| Time-weighted average ketamine infusion rate, mg/h, median (IQR) |  |  |  |  |  |  |
| ED encounter | - | 50.9 (50.9, 50.9) | 0.99 (0.985, 0.995) | - | - | 1 (0.99, 26) |
| Pre-DEX | - | 0 (0, 0) | 0.995 (0.993, 0.998) | - | - | 0.99 (0.495, 0.995) |
| Post-DEX | - | 50.9 (50.9, 50.9) | 0.985 (0.973, 0.998) | - | - | 1.01 (0.985, 26) |
| **Fentanyl** |  |  |  |  |  |  |
| Bolus-dose fentanyl, n (%) |  |  |  |  |  |  |
| ED encounter | 6 (9.4%) | 19 (30.2%) | 11 (20%) | 26 (50%) | 28 (32.6%) | 90 (28.1%) |
| Pre-DEX | 4 (6.3%) | 16 (25.4%) | 10 (18.2%) | 20 (38.5%) | 19 (22.1%) | 69 (21.6%) |
| Post-DEX | 3 (4.7%) | 7 (11.1%) | 5 (9.1%) | 15 (28.8%) | 17 (19.8%) | 47 (14.7%) |
| Bolus-dose fentanyl dose, mg, median (IQR) |  |  |  |  |  |  |
| ED encounter | 113 (100, 181) | 150 (100, 200) | 350 (175, 425) | 150 (100, 288) | 175 (100, 300) | 150 (100, 300) |
| Pre-DEX | 75.0 (12.5, 175) | 100 (75.0, 150) | 150 (100, 200) | 100 (50, 188) | 100 (0, 200) | 100 (50, 200) |
| Post-DEX | 50 (0, 119) | 0 (0, 62.5) | 0 (0, 325) | 50 (0, 100) | 50 (0, 100) | 50 (0, 100) |
| Fentanyl infusion, n (%) |  |  |  |  |  |  |
| ED encounter | 1 (1.6%) | 11 (17.5%) | 7 (12.7%) | 16 (30.8%) | 31 (36%) | 66 (20.6%) |
| Pre-DEX | 0 (0%) | 9 (14.3%) | 6 (10.9%) | 13 (25.0%) | 16 (18.6%) | 44 (13.8%) |
| Post-DEX | 1 (1.6%) | 9 (14.3%) | 7 (12.7%) | 16 (30.8%) | 31 (36%) | 64 (20%) |
| Time-weighted average fentanyl infusion rate, mg/h, median (IQR) |  |  |  |  |  |  |
| ED encounter | 75 (75, 75) | 78.2 (55.3, 111) | 108 (72.4, 145) | 97.8 (66.5, 118) | 92.9 (51.2, 133) | 93.7 (55.2, 129) |
| Pre-DEX | 0 (0, 0) | 79.1 (51.8, 104) | 67.6 (57.7, 110) | 53.4 (40.7, 81.6) | 50 (0, 101) | 54.6 (0, 100) |
| Post-DEX | 75 (75, 75) | 81.3 (49.7, 138) | 100 (68.9, 156) | 98 (76.7, 158) | 94.6 (51.2, 145) | 95.2 (51, 156) |
| **Phenobarbital** |  |  |  |  |  |  |
| Phenobarbital, n (%) |  |  |  |  |  |  |
| ED encounter | 25 (39.1%) | 2 (3.2%) | 0 (0%) | 1 (1.9%) | 3 (3.5%) | 31 (9.7%) |
| Pre-DEX | 22 (34.4%) | 2 (3.2%) | 0 (0%) | 1 (1.9%) | 2 (2.3%) | 27 (8.4%) |
| Post-DEX | 6 (9.4%) | 0 (0%) | 0 (0%) | 0 (0%) | 1 (1.2%) | 7 (2.2%) |
| Phenobarbital dose, mg, median (IQR) |  |  |  |  |  |  |
| ED encounter | 750 (520, 990) | 1010 (635, 1390) | - | 920 (920, 920) | 390 (325, 525) | 661 (520, 977) |
| Pre-DEX | 730 (390, 930) | 1010 (635, 1390) | - | 920 (920, 920) | 260 (130, 460) | 661 (325, 925) |
| Post-DEX | 0 (0, 0) | 0 (0, 0) | - | 0 (0, 0) | 0 (0, 195) | 0 (0, 0) |

**Supplementary Table S4. Additional data.** EWD = ethanol withdrawal, AP-S = acute poisoning by sympathomimetics, AP-M = acute poisoning by multiple substance classes, AP-E = acute poisoning by ethanol, IQR = interquartile range, ED = emergency department. Dose data for the ED encounter, pre-DEX, and post-DEX phases are given for the population of patients who received the sedative in question at any time during the ED course. Some patients may have a zero dose of the sedative for a given sub-phase (e.g. a patient who received diazepam only pre-DEX would have a dose of zero milligrams of diazepam for the post-DEX phase). *Excludes 91 patients who did not receive an EKG in the ED and 2 patients with an EKG that was performed but missing from the chart.
