# Supplementary material for: Dexmedetomidine in The Treatment of Toxicologic Conditions in The Emergency Department: A Dual-Center Retrospective Observational Cohort Study
Source: J Med Toxicol. 2026 Jul 10;22(3):364–74. doi: 10.1007/s13181-026-01145-5 (PMC13407800; doi:10.1007/s13181-026-01145-5)
Supplement: Supplementary file 6 — Supplementary file6 (DOCX 21 KB) [file 13181_2026_1145_MOESM6_ESM.docx]

**APPENDIX: Intubation Sensitivity Analysis**

|  | **Intubated**  **Pre-DEX**  **(n = 120)** | **Intubated Post-DEX**  **(n = 59)** | **Not Intubated Post-DEX**  **(n = 141)** |
| --- | --- | --- | --- |
| **Candidate Factors for Multivariable Model** | | | |
| **Blood ethanol, mg/dL**, **median (IQR)*** | 23.5 (0, 237) | 0 (0, 18.0) | 0 (0, 125) |
| Blood ethanol not tested, n (%) | 24 (20%) | 18 (30.5%) | 60 (42.6%) |
| Blood ethanol detectable, n (%) | 51 (42.5%) | 12 (20.3%) | 34 (24.1%) |
| Blood ethanol undetectable, n (%) | 45 (37.5%) | 29 (49.2%) | 47 (33.3%) |
| **Any benzodiazepine, n (%)** | 35 (29.2%) | 48 (81.4%) | 106 (75.2%) |
| **Any antipsychotic, n (%)** | 24 (20%) | 40 (67.8%) | 74 (52.5%) |
| **Any ketamine, n (%)** | 8 (6.7%) | 17 (28.8%) | 28 (19.9%) |
| **Other Variables (Descriptive)** | | | |
| **Study site, n (%)** |  |  |  |
| BJH | 86 (71.7%) | 37 (62.7%) | 89 (63.1%) |
| HCMC | 34 (28.3%) | 22 (37.3%) | 52 (36.9%) |
| **Tox condition, n (%)** |  |  |  |
| Withdrawal, ethanol | 15 (12.5%) | 8 (13.6%) | 41 (29.1%) |
| Acute poisoning, sympathomimetics | 18 (15.0%) | 13 (22.0%) | 32 (22.7%) |
| Acute poisoning, multiple classes | 26 (21.7%) | 9 (15.3%) | 20 (14.2%) |
| Acute poisoning, ethanol | 28 (23.3%) | 8 (13.6%) | 16 (11.3%) |
| Other conditions | 33 (27.5%) | 21 (35.6%) | 32 (22.7%) |
| **Pre-DEX RASS, median (IQR)** | 4.00 (4.00, 4.00) | -0.500 (-2.00, 1.00) | 0 (-1.00, 0.625) |
| Missing, n (%) | 58 (98.3%) | 63 (52.5%) | 133 (94.3%) |
| **Pre-DEX GCS, median (IQR)** | 14.0 (13.0, 15.0) | 7.00 (3.00, 12.5) | 14.0 (12.0, 15.0) |
| Missing, n (%) | 7 (11.9%) | 20 (16.7%) | 26 (18.4%) |
| **Any ED vasopressor, n (%)** | 35 (29.2%) | 11 (18.6%) | 9 (6.4%) |
| Any ED infusion vasopressor, n (%) | 31 (25.8%) | 8 (13.6%) | 9 (6.4%) |
| Maximum norepinephrine equivalent rate, mcg/kg/min, median (IQR) | 0.07 (0.05, 0.13) | 0.25 (0.12, 0.66) | 0.08 (0.05, 0.12) |
| Any ED bolus vasopressor, n (%) | 14 (11.7%) | 7 (11.9%) | 5 (3.5%) |
| **Any ED NPPV, n (%)** | 14 (11.7%) | 17 (28.8%) | 37 (26.2%) |
| **Any IV fluid resuscitation, n (%)** | 104 (86.7%) | 44 (74.6%) | 109 (77.3%) |
| **ED disposition, n (%)** |  |  |  |
| ICU | 106 (88.3%) | 58 (98.3%) | 111 (78.7%) |
| Stepdown/OU | 1 (0.8%) | 0 | 2 (1.4%) |
| Floor | 5 (4.2%) | 0 | 9 (6.4%) |
| Discharged | 8 (6.7%) | 1 (1.7%) | 18 (12.8%) |
| Expired | 0 | 0 | 1 (0.7%) |
| **Hospital mortality, n (%)** | 3 (2.5%) | 2 (3.4%) | 3 (2.1%) |

**Supplementary Table S5. Patient characteristics by intubation status (sensitivity analysis extending intubation window to 24 hours after DEX initiation).** *Excludes patients not tested for ethanol. DEX = dexmedetomidine, IQR = interquartile range, BJH = Barnes-Jewish Hospital, HCMC = Hennepin County Medical Center, ED = emergency department, NPPV = non-invasive positive pressure ventilation.
