# Supplementary material for: Dexmedetomidine in The Treatment of Toxicologic Conditions in The Emergency Department: A Dual-Center Retrospective Observational Cohort Study
Source: J Med Toxicol. 2026 Jul 10;22(3):364–74. doi: 10.1007/s13181-026-01145-5 (PMC13407800; doi:10.1007/s13181-026-01145-5)
Supplement: Supplementary file 7 — Supplementary file7 (DOCX 34 KB) [file 13181_2026_1145_MOESM7_ESM.docx]

**APPENDIX 7: Detailed Statistical Analysis and Model Specifications**

**Methods**

*Main Analysis*

We used logistic regression to explore associations between candidate predictors (blood ethanol concentration [detected, undetected, not tested], administration of any benzodiazepine [yes/no], administration of any antipsychotic [yes/no], and administration of ketamine [yes/no]) and our outcome, intubation after DEX administration in the ED. We excluded patients intubated prior to receiving DEX to ensure only patients at risk for the outcome were included in this analysis (n = 200). To minimize reverse causality, only pre-intubation medication administration was considered among patients who were intubated. Among patients who were not intubated, receipt of the medications at any point during the ED stay was considered exposure. In addition to the main analysis model, we conducted four sensitivity analyses.

*Sensitivity Analyses*

1. **Including Random Effect for Study Site**

Sensitivity analysis 1 included the same candidate predictors as the main analysis model with an additional random intercept for study site. We assessed the contribution of fixed and random effects using the marginal R² (variance explained by fixed effects alone), conditional R² (variance explained by the full model, including random effects), and the intraclass correlation coefficient (ICC), which quantifies the proportion of total outcome variance attributable to between-site differences.

**2. Testing blood alcohol concentration as a continuous variable and dropping patients who were not tested**

Sensitivity analysis 3 included blood alcohol concentration as a continuous variable and excluded patients not tested for alcohol. Given that many patients had a blood alcohol concentration of 0, the variable was scaled per 100 mg/dL, such that model coefficients represent the change in odds of intubation for each 100 mg/dL increase.

**3. Taking into account post-ED intubations in the first 24 hours after DEX initiation.**

Sensitivity analysis 4 incorporated post-ED intubations in the first 24 hours. As such, we redefined our outcome variable to include the following three levels: (1) Not intubated in ED nor in the 24 hours after DEX initiation, (2) Intubated in ED prior to DEX administration (i.e., the group not at risk), and (3) Intubated in ED after DEX administration or intubated in the 24 hours after DEX initiation.

**Results:**

| **Table 1. Descriptive Statistics for Regression Covariates** | | | |
| --- | --- | --- | --- |
|  | **Intubated in ED after DEX administration (N=43)** | **Not intubated in ED (N=157)** | **Overall (N=200)** |
| **Blood Alcohol Result** |  |  |  |
| Negative | 22 (51.2%) | 54 (34.4%) | 76 (38.0%) |
| Not tested | 14 (32.6%) | 64 (40.8%) | 78 (39.0%) |
| Positive | 7 (16.3%) | 39 (24.8%) | 46 (23.0%) |
| **Received Benzodiazepines** |  |  |  |
| Yes | 38 (88.4%) | 116 (73.9%) | 154 (77.0%) |
| No | 5 (11.6%) | 41 (26.1%) | 46 (23.0%) |
| **Received Antipsychotics** |  |  |  |
| Yes | 32 (74.4%) | 82 (52.2%) | 114 (57.0%) |
| No | 11 (25.6%) | 75 (47.8%) | 86 (43.0%) |
| **Received Ketamine** |  |  |  |
| Yes | 10 (23.3%) | 35 (22.3%) | 49 (24.5%) |
| No | 33 (76.7%) | 122 (77.7%) | 155 (77.5%) |
| **Study Site** |  |  |  |
| BJH | 22 (51.2%) | 104 (66.2%) | 126 (63.0%) |
| HCMC | 21 (48.8%) | 53 (33.8%) | 74 (37.0%) |
| **Pre-DEX Median GCS** | 14.0 (12.0, 15.0) | 14.0 (13.0, 15.0) | 14.0 (12.3, 15.0) |
| Missing | 27 (17.2%) | 6 (14.0%) | 33 (16.5%) |

**Main Analysis**

The main adjusted analysis model found a significant association between pre-intubation antipsychotic administration and intubation (Table 2).

| **Table 2.** **Main Analysis Logistic Regression Results (n = 200)** | | |
| --- | --- | --- |
| **Variable Name** | **Adjusted OR (95%CIs)** | **Unadjusted OR (95%CI)** |
| **Blood Alcohol** |  |  |
| Negative (Ref) | 1.0 | 1.0 |
| Positive | 0.48 (0.17, 1.21) | 0.44 (0.16, 1.09) |
| Not tested | 0.68 (0.30, 1.49) | 0.54 (0.25, 1.14) |
| **Benzodiazepines** | 2.24 (0.87, 6.96) | 2.69 (1.07, 8.20)* |
| **Antipsychotics** | 2.48 (1.17, 5.57)* | 2.66 (1.29, 5.87)* |
| **Ketamine** | 0.97 (0.40, 2.18) | 1.06 (0.46, 2.29) |

**Sensitivity Analysis # 1: Add Random Effect for Study Site**

The multilevel logistic regression yielded a marginal R² of 0.126 and a conditional R² of 0.147, indicating that fixed effects explained 12.6% of the variance and the full model (including a random intercept for site) explained 14.7%. The adjusted ICC was 0.024, suggesting that ~2.4% of the variance was attributable to between-site differences. The multilevel logistic regression results were not meaningfully different from the primary model (Table 3).

| **Table 3. Sensitivity Analysis 1: Multilevel logistic Regression Results with Random Intercept for Study Site (n = 200)** | | |
| --- | --- | --- |
| **Variable Name** | **Adjusted OR (95%CIs)** | **Unadjusted OR (95%CI)** |
| **Blood Alcohol** |  |  |
| Negative (Ref) | 1.0 | 1.0 |
| Positive | 0.42 (0.15, 1.17) | 0.44 (0.16, 1.09) |
| Not tested | 0.57 (0.24, 1.40) | 0.54 (0.25, 1.14) |
| **Benzodiazepines** | 1.95 (0.66, 5.72) | 2.69 (1.07, 8.20)* |
| **Antipsychotics** | 2.52 (1.15, 5.51)* | 2.66 (1.29, 5.87)* |
| **Ketamine** | 1.01 (0.43, 2.38) | 1.06 (0.46, 2.29) |

**Sensitivity Analysis #2: Treating Alcohol Concentration as a continuous variable.**

Similar to when we adjusted for illness severity (GCS), the association between antipsychotics and intubation was no longer significant after treating alcohol concentration as a continuous variable. The association between benzodiazepines and intubation likewise became significant with a wide confidence interval. The estimate and confidence intervals were similar in the unadjusted analysis, suggesting this change may have been due to the change in sample from dropping patients not tested for alcohol (Table 5).

| **Table 5. Sensitivity Analysis 2: Logistic Regression Results Treating EtOH as a Continuous Variable (n = 122)** | | |
| --- | --- | --- |
| **Variable Name** | **Adjusted OR (95%CIs)** | **Unadjusted OR (95%CI)** |
| **Blood Alcohol** | 0.75 (0.46, 1.12) | 0.75 (0.45, 1.10) |
| **Benzodiazepines** | 3.85 (1.01, 25.40)* | 3.94 (1.06, 25.66)* |
| **Antipsychotics** | 2.62 (0.99, 7.88) | 2.65 (1.04, 7.72)* |
| **Ketamine** | 1.05 (0.36, 2.81) | 1.39 (0.51, 3.53) |

**Sensitivity Analysis #3: Considering intubation in the 24-hour post-DEX.**

| **Table 6. Descriptive Statistics for Sensitivity Analysis 3: Considering Intubation in the 24-hours Post-DEX Initiation** | | | |
| --- | --- | --- | --- |
|  | **Intubated in ED after DEX administration or intubated in the 24 hours after DEX initiation (N=59)** | **Not intubated in ED nor in the 24 hours post DEX (N=141)** | **Overall (N=200)** |
| **Alcohol Concentration – Median (IQR)** | 0 (0, 18.0) | 0 (0, 125) | 0 (0, 107) |
| **Alcohol Concentration – n (%)** |  |  |  |
| Undetectable | 29 (49.2%) | 47 (33.3%) | 76 (38.0%) |
| Detectable | 12 (20.3%) | 34 (24.1%) | 46 (23.0%) |
| Not tested | 18 (30.5%) | 60 (42.6%) | 78 (39.0%) |
| **Benzodiazepines** |  |  |  |
| Yes | 48 (81.4%) | 106 (75.2%) | 154 (77.0%) |
| No | 11 (18.6%) | 35 (24.8%) | 46 (23.0%) |
| **Antipsychotics** |  |  |  |
| Yes | 40 (67.8%) | 74 (52.5%) | 114 (57.0%) |
| No | 19 (32.2%) | 67 (47.5%) | 86 (43.0%) |
| **Ketamine** |  |  |  |
| Yes | 17 (28.8%) | 28 (19.9%) | 45 (22.5%) |
| No | 42 (71.2%) | 113 (80.1%) | 155 (77.5%) |

The sensitivity analysis 3 adjusted model found no significant associations between any covariates tested and intubation in the 24 hours post-DEX initiation (Table 2).

| **Table 7. Sensitivity Analysis 3: Logistic Regression Results When Considering Intubation in the 24-hours Post-DEX Initiation (n = 200)** | | |
| --- | --- | --- |
| **Variable Name** | **Adjusted OR (95%CIs)** | **Unadjusted OR (95%CI)** |
| **Blood Alcohol** |  |  |
| Negative (Ref) | 1.0 | 1.0 |
| Positive | 0.58 (0.25, 1.30) | 0.57 (0.25, 1.26) |
| Not tested | 0.55 (0.26, 1.12) | 0.49 (0.24, 0.97)* |
| **Benzodiazepines** | 1.25 (0.58, 2.83) | 1.44 (0.69, 3.19) |
| **Antipsychotics** | 1.68 (0.88, 3.29) | 1.91 (1.02, 3.67)* |
| **Ketamine** | 1.57 (0.76, 3.22) | 1.63 (0.80, 3.27) |
